# Dietary non-enzymatic antioxidant capacity and risk of breast cancer: the Swedish National March Cohort

**DOI:** 10.1186/s12885-025-14658-z

**Published:** 2025-08-13

**Authors:** Daniela Mariosa, Marta Ponzano, Alessandra Grotta, Ylva Trolle Lagerros, Essi Hantikainen, Hans-Olov Adami, Mauro Serafini, Rino Bellocco, Weimin Ye

**Affiliations:** 1 International Agency for Research on Cancer (IARC/WHO), Genomic Epidemiology Branch, Lyon, France; 2 Department of Statistics and Quantitative Methods, University of Milano-Bicocca, Milan, Italy; 3 Department of Health Sciences, University of Genoa, Genoa, Italy; 4 Department of Public Health Sciences, Stockholm University, Stockholm, Sweden; 5 Centre for Health Equity Studies, Stockholm University/Karolinska Institutet, Stockholm, Sweden; 6 Department of Medicine, Karolinska Institutet, Huddinge, Sweden; 7 Institute for Biomedicine (Affiliated to the University of Lübeck), Eurac Research, Bozen, Italy; 8 Clinical Effectiveness Group, Institute of Health and Society, University of Oslo, Oslo, Norway; 9 Department of Medical Epidemiology and Biostatistics, Karolinska Institutet, Box 281, Stockholm, SE-17177 Sweden; 10 Functional Food and Metabolic Stress Prevention Lab, Department of Biosciences and Technologies for Agriculture, Food and Environment, University of Teramo, Teramo, Italy

**Keywords:** Diet, Antioxidants, Breast cancer, Nutrition, Cohort study

## Abstract

**Background:**

Total dietary antioxidant capacity has been associated with a lower risk of breast cancer​, but the supporting evidence is limited. We investigated the association between dietary Non-Enzymatic Antioxidant Capacity (NEAC), measuring the total antioxidant potential of the diet, and the risk of breast cancer.

**Methods:**

We followed 24,950 women recruited into the Swedish National March Cohort through record linkages to Swedish health registries from 1997 until 2016. Total NEAC was computed based on the baseline 96-item food frequency questionnaire. Three measures of dietary NEAC were assessed: total NEAC, NEAC from fruits and vegetables and NEAC from grains. We fitted multivariable Cox proportional hazards regression models to compute hazard ratios (HRs) and 95% confidence intervals (CIs) to quantify the association between dietary NEAC and risk of overall, as well as pre- and post-menopausal breast cancer.

**Results:**

During a median follow-up of 19.2 years, 1142/24,950 women were diagnosed with breast cancer (136/10,826 pre- and 975/21,152 post-menopausal). Findings indicated a trend in the association between total NEAC and the hazard of overall breast cancer (highest vs. lowest quartile: adjusted HR = 0.85, 95% CI 0.69-1.04; *p*-value for Wald test = 0.138, *p*-value for trend = 0.048). The association was more evident for post-menopausal breast cancer (HR = 0.76, 95% CI 0.60-0.96; *p*-value for trend = 0.010). However, when missing data were imputed, the magnitude of the association was found to be weaker in terms of hazard reduction and no statistically significant associations were observed but the direction of the associations remained consistent (overall: HR = 0.89 (0.74-1.08), *p*-value for trend = 0.091; post-menopausal: HR = 0.84 (0.69-1.03), p-value for trend = 0.057). When distinguishing NEAC based on food sources, breast cancer hazard was inversely associated with NEAC from fruits and vegetables (HR = 0.79, 95% CI 0.64-0.97; *p*-value for trend = 0.019), but not with NEAC from grains (HR = 1.05, 95% CI 0.86-1.29; *p*-value for trend = 0.630).

**Conclusion:**

These results suggest an inverse association between dietary total antioxidant capacity and the risk of breast cancer, particularly in post-menopausal women, which seems to be driven by the consumption of fruits and vegetables. However, sensitivity analysis on imputed covariates did not fully confirm our findings, indicating the need for future confirmatory research.

**Supplementary Information:**

The online version contains supplementary material available at 10.1186/s12885-025-14658-z.

## Introduction

Breast cancer is the most common cancer worldwide accounting for 24% of all incident cancers among women in 2018, with an incidence rate of 90 per 100,000 person-years in Northern Europe [1, 2]. Although several causal risk factors for breast cancer are established, few are easily accessible for primary prevention [3]. Dietary antioxidant intake is modifiable and influences processes involved in carcinogenesis such as oxidative and inflammatory stress and angiogenesis [4]. Methods have been developed to estimate total antioxidant capacity in plasma derived from foods [5, 6], also referred to as Non-Enzymatic Antioxidant Capacity (NEAC) [7, 8]. NEAC was originally chosen as the best method to measure dietary antioxidant intake because antioxidant capacity in plasma from diet provides an overall assessment of the antioxidant activity from diet, including also potential synergistic activities, and not of the level of single antioxidants. Moreover, we have shown how dietary antioxidant intake is associated with plasma levels of antioxidants, suggesting the importance of a high antioxidant intake to maintain physiological levels of plasma antioxidant, avoiding oxidative stress conditions [9]. Our study on gastric cancer was the first epidemiological study to assess the effect of dietary NEAC estimated from information on food and beverage consumption [10]. This approach has now been utilized in many epidemiological [11, 12] as well as in intervention studies [13, 14]. In the only prospective study, to date, on the association between dietary NEAC and breast cancer high dietary NEAC was associated with a lower risk of breast cancer in post-menopausal women [15]. Dietary antioxidant intake may contribute to the protective effects of healthy diet on breast cancer risk, for which there is more evidence in postmenopausal women than in premenopausal women [16]. Therefore, additional evidence from prospective studies is critical for confirming that NEAC is inversely associated with the risk of post-menopausal breast cancer and for investigating if NEAC may be associated with the risk of pre-menopausal breast cancer, which has not been previously assessed in a prospective study.

In the present analysis, we studied the association between NEAC and the risk for breast cancer among women in the Swedish National March Cohort (SNMC). We estimated the association for breast cancer overall and further evaluated associations for pre-menopausal and post-menopausal breast cancer specifically.

## Methods

### Study cohort

The Swedish National March Cohort (SNMC) was established in conjunction with a 4-day national fund-raising event arranged in 1997 by the Swedish Cancer Society in almost 3600 cities and villages around Sweden [17]. Due to the nature of the event, the number of participants could not be assessed in detail. All participants (women and men above the age of 18) were invited to fill out a 32-page questionnaire once with detailed questions about demographics, medical history, anthropometric measures, physical activity [18], history of smoking and alcohol drinking. In particular, the questionnaire included 106 questions that collected comprehensive information on dietary habits [19]. A total of 43,880 participants completed the baseline questionnaire and entered into the study. Participants provided their individual and unique 10-digit national registration numbers, but 17 subjects with an incorrect national registration number (inconsistent or non-existent) were excluded [17]. In the overall cohort, median (10–90 percentiles) age was 52 (23-71) years. Participants were 36% males and 64% females but, in our study, only women were included. Further details on the cohort profile have been previously published [17].

The cohort was followed-up through linkages to nationwide Swedish Registers using the national registration numbers (NRNs), unique identification numbers assigned to all Swedish residents [20]. The Register of the Total Swedish Population maintained by the government agency Statistics Sweden provides information about dates of emigration and death since 1968 [21]. The Swedish Cancer Register, established in 1958, records all cancer diagnoses, including information on anatomic subsite, histology and histopathology [22].

**Fig. 1:**
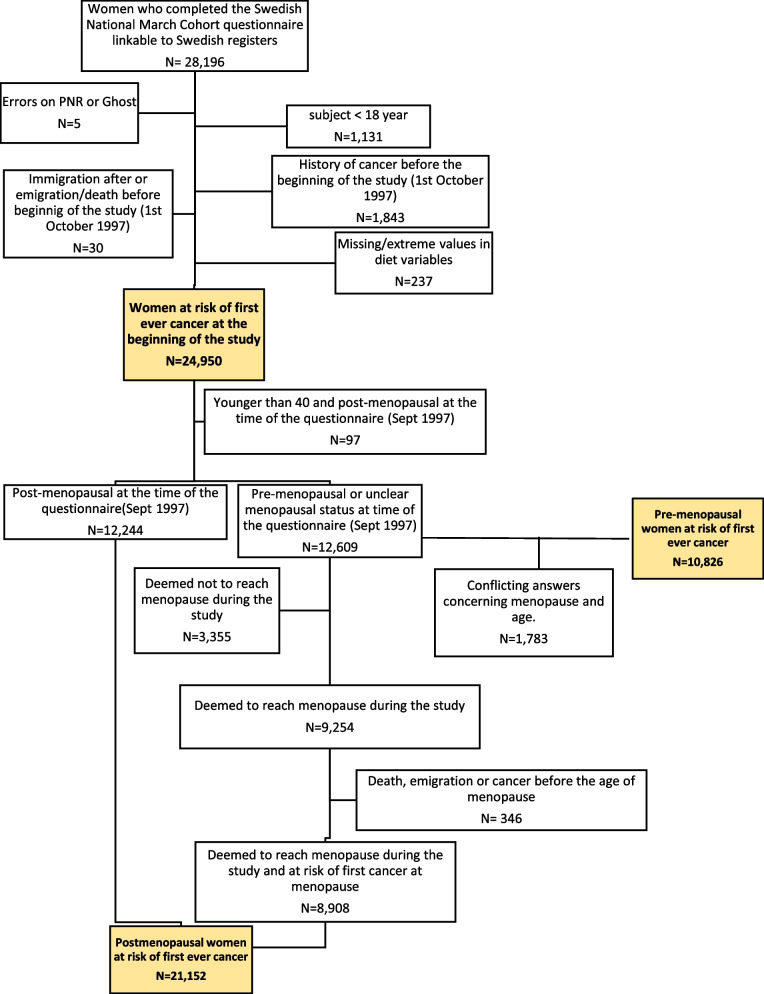
Flow diagram of the number of women of the Swedish National March Cohort who were eligible for inclusion in the analyses. Women *deemed to reach menopause* included women that underwent bilateral oophorectomy or reached presumed time of natural menopause (age 50 if age at enrolment <50, or age 60 if age at enrolment ≥50)

Among the 28,196 women of the SNMC, 24,950 were at risk of first ever cancer at cohort recruitment and therefore eligible for inclusion in the present analysis (Fig. 1).

### Non-enzymatic antioxidant capacity

The participants answered a semi-quantitative 96-item food frequency questionnaire (FFQ) with eight predetermined response categories of consumption, ranging from “never” to “three times daily, or more”. This FFQ is a slightly abbreviated version of a validated 96-item FFQ questionnaire [19, 23], which in its original form requested the participants to also choose from three different portion sizes (small, medium and large) for each item. In this version, average daily intake for every food item was created for each subject by multiplying the frequency of consumption of specific food items considering standard, item-specific portion sizes. Within the FFQ standard portion sizes were displayed for each item to aid estimation of their daily intake. Out of the 96 items, 80 were used to estimate nutrient intake, excluding items related to food preparation. We calculated daily total energy and nutrient intakes using the Swedish food composition database [24]. It is managed by the National Food Agency and covers nutrient information and standard portion sizes on more than 2300 foods and dishes on the Swedish market [25].

NEAC can be measured through different chemical assays, such as the Ferric-Reducing Antioxidant Potential (FRAP), Total Radical-Trapping Antioxidant Parameter (TRAP) and TEAC (plasma Trolox Equivalent Antioxidant Capacity). The FRAP and TRAP assays evaluate the chain-breaking antioxidant potential, whereas the TEAC assay measures the ability of antioxidants to quench a radical cation (ABTS·+) in both lipophilic and hydrophilic environments [5]. For our study we calculated NEAC from foods in terms of FRAP, which, more specifically is based on the reduction of ferric iron (Fe^3+)^ to ferrous iron (Fe^2+^) [5]. As previously described [26], NEAC daily intake was calculated for each food by multiplying food-specific intake by food-specific FRAP expressed as mmol Fe^2+^ equivalents per 1-kg fresh weight of single foods [5, 6] which was derived from published databases. Total daily intake of NEAC for each woman was then calculated by summing FRAP from all food contributors. The assessment of dietary NEAC using FRAP, TRAP and TEAC has previously been validated and compared with plasma response in Swedish women [9].

NEAC values were available for 66 out of the 80 items from the FFQ used for nutrient estimation, resulting in a certain degree of measurement error into the main exposure variable. Food items or groups without information on NEAC contained mainly animal products (meat, dairy products), sweets and pastries, which are low in antioxidant content. When we found several matches in the NEAC database for a food item that was more generally defined in the questionnaire, e.g. consumption frequency of apple and pear was asked in the same question, we used market consumption data to compute NEAC as weighted mean of all the suitable values to estimate NEAC for the combined item. We disregarded NEAC from coffee consumption, since it remains unclear whether the main contributors to the in vitro antioxidant capacity of coffee are absorbed efficiently and if the same antioxidant activity is exerted in vivo. However, we adjusted for coffee consumption in our analyses.

We additionally considered NEAC from two main food subgroups to investigate whether the association with risk varied depending on the source of NEAC. We computed daily NEAC from the 16 questions addressing vegetables and fruit intake, and from the 10 questions addressing grain consumption. Moreover, due to the relevant contribution of tea consumption to dietary NEAC in our cohort, the association between tea-derived NEAC and breast cancer risk was explored.

### Ascertainment of breast cancer cases

Cases of breast cancer were defined as first-ever diagnosis (ICD-7 170) in the Cancer Register during follow-up. We excluded 6 cases with a breast cancer diagnosis found in the Cause of Death Register only, since they were never reported to the Cancer Register. Because menopausal status at the time of the cancer diagnosis was not available, we inferred this variable based on the age at cancer diagnosis and on the menopausal status at recruitment. To define menopausal status, we combined four items in the questionnaire: age and presence of menstruation at baseline, age at menopause (for women already in menopause) and, if menstruation did not cease spontaneously, date of oophorectomy. Based on this information and on the mean and median age at menopause in Sweden (mean (SD): 50.2 (4.4); median (interquartile range, IQR): 50.0 (48.0-53.0)) [27], we inferred menopause also for women who were not in menopause at recruitment as date of oophorectomy, if occurring first, or when they subsequently turned 50 (age at enrollment <50) or 60 (age at enrollment ≥50). Depending on the inferred menopause age, women could thus contribute to both the pre- and post-menopausal analyses or just to one of them. When age at enrollment, presence of menstruation and age at menopause were conflicting, we excluded these women from the analyses of menopausal status. Moreover, 97 women younger than 40 and post-menopausal at the time of the questionnaire (premature menopause) were excluded from the pre- and post-menopausal analyses because these women may have either peculiar characteristics that led to premature menopause or have incorrectly reported to be postmenopausal [27]. Our final analytic samples consisted of *n* = 10,826 pre-menopausal and *n* = 21,152 post-menopausal women. A detailed description of the sample selection is provided in Fig. 1.

### Statistical analyses

Women were followed from enrollment in 1997 until diagnosis of breast cancer. Follow-up was censored at the date of other malignant cancers, death, emigration out of Sweden, or end of follow-up (December 31, 2016), whichever came first. Cox proportional hazards regression models with age as the underlying time scale were fitted to estimate hazard ratios (HRs) with 95% confidence intervals (CIs). We adjusted NEAC for total energy intake by using the nutrient residual model [28]. NEAC was then entered into the models as a categorical variable and Wald test for the significance of the overall effect of dietary NEAC was performed. NEAC categories were defined based on quartiles, with the lowest quartile used as reference category. Categorization could thus differ in pre- and post-menopausal subsamples since quartiles were recalculated in each subsample. The *p*-value for trend was calculated from a separate model including a semi-continuous variable (median of each category of NEAC) derived from the categorical variable. In addition, a logarithmic transformation of dietary NEAC was also included as a continuous variable. We evaluated the assumption of proportionality of hazards in Cox regression models by testing interaction of the HRs with time using the scaled Schoenfeld residuals. A dose–response relationship was investigated based on restricted cubic splines with 4 knots at the 5th, 35th, 65th and 95th percentiles of the distribution of dietary NEAC.

We examined potential confounding in a causal inference framework using Directed Acyclic Graphs (DAGs) [29]. We identified the following potential confounders: body mass index (BMI) (normal weight: <25 kg/m^2^, overweight: ≥25 and < 30 kg/m^2^, obesity: ≥30 kg/m^2^), physical activity (<31.8, ≥31.8 and < 39.0, ≥39.0 metabolic equivalent of task (MET)-hours per day (METh/day)), cigarette smoking (never, past, current), alcohol drinking (all types of alcoholic beverage: never, low: ≤3 times/month, medium: 1–6 time/week, high: ≥1 time/day), coffee intake (0, 1-2, 3-4, 5 or more cups per day), use of vitamin and mineral supplements (yes, no), level of education (7–9, 10–12 or > 12 years), use of contraceptive pills (yes, no), age at the first menstruation (<15 or ≥ 15 years), number of children (<3 or ≥ 3), treatment for infertility (yes, no), hormone replacement therapy (yes, no), and total energy intake (Supplementary Fig. 1).

A multivariable adjustment for all the identified potential confounders was used to estimate the adjusted association between total daily NEAC intake and breast cancer risk overall and by menopausal status. In the multivariable-adjusted models we excluded women with missing values in the covariates (i.e., complete-case analysis). Since hormone replacement therapy is only prescribed to post-menopausal women, this variable was only included in the models for post-menopausal breast cancer.

The association between NEAC and breast cancer risk may vary by the use of dietary supplements (yes, no), cigarette smoking (former or never, current) and BMI (normal or overweight, obese), and for post-menopausal women by the use of hormone replacement therapy (yes, no) [29–31]. Therefore, we performed stratified analyses to investigate potential differential associations between NEAC and cancer risk for different levels of these risk factors.

As a sensitivity analysis, missing values in covariates were imputed using multiple imputation by chained equations (10 datasets imputed). A second sensitivity analysis was performed by starting the follow-up two years after the questionnaire date to reduce the influence of reverse causality. Additionally, the main results were replicated restricting follow-up to 10 years. Lastly, we conducted a sensitivity analysis to investigate the association between NEAC and both premenopausal and postmenopausal breast cancer after imputing the age at menopause instead of attributing fixed ages of 50 or 60. In our cohort age of menopause was recoded in categories of age (“<40”, “40-44”, “45-49”, “50-54”, “55-59”, “> = 60”) and we imputed missing values by multiple imputation by chained equations (10 datasets imputed) based on variables which are known to affect menopausal age [32]. After imputation of this ordinal variable, we inferred the age at menopause as the central age per category and as the extreme values for the first and last categories.

All statistical analyses were performed with Stata version 15.1 (Stata Corporation, College Station, TX, USA). All reported probabilities (*p*-values) were two-sided and *p*-values lower than 0.05 were considered statistically significant.

## Results

Among all women at risk of developing breast cancer (*n* = 24,950, median age (IQR) = 50.6 (38.9-61.5); whereof pre-menopausal *n* = 10,826, median age (IQR) = 37.2 (28.8-44.0) and post-menopausal *n* = 21,152, median age (IQR) = 53.5 (44.7-63.5)), the daily dietary NEAC (measured in terms of residual-adjusted FRAP) was on average 9.7 mmol Fe^2+^ equivalents/day. NEAC intake was higher among women who were older, were more educated, had a higher alcohol consumption, were more physically active, used hormone replacement therapy and who were normal weight and non-smokers (Table 1).

**Table 1:** Characteristics of women in the Swedish National March Cohort by category of dietary NEAC

	Dietary NEAC^a^			
	<6.7	6.7-8.9	8.9-11.7	> = 11.7
Overall	*n* = 6238	*n* = 6237	*n* = 6238	*n* = 6237
Premenopausal	(*n* = 2707)	(*n* = 2706)	(*n* = 2707)	(*n* = 2706)
Postmenopausal	(*n* = 5288)	(*n* = 5288)	(*n* = 5288)	(*n* = 5288)
Variables				
Age at entry (years), mean (SD)	49 (15)	50 (15)	50 (15)	51 (15)
Body mass index (kg/m^2^), n (%)				
Normal	3569 (57)	3700 (59)	3906 (63)	4051 (65)
Overweight	1772 (28)	1748 (28)	1633 (26)	1534 (25)
Obese	587 (9)	493 (8)	434 (7)	364 (6)
Missing	310 (5)	296 (5)	265 (4)	288 (5)
Total energy intake (kcal/day), n (%)				
< 1545	1450 (23)	1067 (17)	1019 (16)	1454 (23)
1545-1782	1304 (21)	1230 (20)	1175 (19)	1281 (21)
1782- 2013	1247 (20)	1245 (20)	1293 (21)	1205 (19)
2013 - 2310	1176 (19)	1311 (21)	1317 (21)	1186 (19)
> 2310	1061 (17)	1384 (22)	1434 (23)	1111 (18)
Education (years), n (%)				
7-9	2691 (43)	2507 (40)	2250 (36)	1968 (32)
10-12	1971 (32)	1914 (31)	1893 (30)	1630 (26)
≥ 12	1450 (23)	1711 (27)	1975 (32)	2504 (40)
Missing	126 (2)	105 (2)	120 (2)	135 (2)
Smoking, n (%)				
Never	3598 (58)	3789 (61)	3937 (63)	4011 (64)
Former	1449 (23)	1501 (24)	1469 (24)	1480 (24)
Current	705 (11)	498 (8)	449 (7)	338 (5)
Missing	486 (8)	449 (7)	383 (6)	408 (7)
Alcohol (g/month), n (%)				
Never	919 (15)	748 (12)	677 (11)	680 (11)
< 108	2069 (33)	1842 (30)	1721 (28)	1682 (27)
108-303	1795 (29)	1852 (30)	1913 (31)	1712 (27)
> 303	1438 (23)	1786 (29)	1915 (31)	2154 (35)
Missing	17 (0.3)	9 (0.1)	12 (0.2)	9 (0.1)
Coffee (cups/day), n (%)				
0	573 (9)	645 (10)	698 (11)	1378 (22)
1-2	1263 (20)	1599 (26)	2144 (34)	2450 (39)
3-4	2900 (46)	2839 (46)	2582 (41)	1722 (28)
≥ 5	1502 (24)	1154 (19)	814 (13)	687 (11)
Physical Activity (METh/day), n (%)				
< 31.8	1944 (31)	1922 (31)	1822 (29)	1835 (29)
31.8-39.0	1812 (29)	1841 (30)	1912 (31)	1955 (31)
≥ 39.0	1802 (29)	1883 (30)	1938 (31)	1898 (30)
Missing	680 (11)	591 (9)	566 (9)	549 (9)
Vitamin and mineral supplement use, n (%)				
Yes	2020 (32)	2113 (34)	2189 (35)	2149 (34)
No	4023 (64)	3953 (63)	3881 (62)	3921 (63)
Missing	195 (3)	171 (3)	168 (3)	167 (3)
Contraceptive pill use, n (%)				
Yes	4024 (65)	4006 (64)	4057 (65)	4101 (66)
No	2088 (33)	2107 (34)	2068 (33)	2028 (33)
Missing	126 (2)	124 (2)	113 (2)	108 (2)
Hormone replacement therapy, n (%)				
Yes	1493 (24)	1622 (26)	1681 (27)	1856 (30)
No	4579 (73)	4462 (72)	4419 (71)	4242 (68)
Missing	166 (3)	153 (2)	138 (2)	139 (2)
Age at menarche (years), n (%)				
< 15	5134 (82)	5037 (81)	5090 (82)	5090 (82)
≥ 15	961 (15)	1080 (17)	1027 (16)	1026 (16)
Missing	143 (2)	120 (2)	121 (2)	121 (2)
Number of children, n (%)				
< 3	5707 (91)	5758 (92)	5774 (93)	5746 (92)
≥ 3	500 (8)	449 (7)	441 (7)	466 (7)
Missing	31 (0.5)	30 (0.5)	23 (0.4)	25 (0.4)
Treated for childlessness, n (%)				
Yes	319 (5)	351 (6)	303 (5)	340 (5)
No	5851 (94)	5812 (93)	5871 (94)	5834 (94)
Missing	68 (1)	74 (1)	64 (1)	63 (1)

*Abbreviations*: *MET* Metabolic equivalent of task, *SD* Standard deviation

^a^NEAC was measured with the NEAC assay expressed in mmol Fe2+ equivalents/day

Figure 1 shows the flow diagram of the number of women eligible for the overall, pre- and post-menopausal analysis. Specifically, of the 12,609 women who were pre-menopausal or reported unclear menopausal status at time of the questionnaire, a total of 8908 (71%) were included in the post-menopausal subsample, after inferring their menopause date as described in the Methods section. In particular, the 12,609 women with missing menopause date reported a median (IQR) age at recruitment of 39.3 (30.1-46.2) years; a total of 1330 (11%) were assigned menopause age of 60 years and 11,279 (89%) age of 50 years.

Overall, food groups that contributed most to dietary NEAC were fruit and vegetables (23%), grain (19%), tea (28%), chocolate (9%) and alcohol (4%) across the overall study sample. In pre-menopausal women the most contributing food groups were fruit and vegetables (22%), grain (17%), tea (32%), chocolate (10%) and alcohol (3%) and for the post-menopausal women, fruit and vegetables (24%), grain (19%), tea (28%), chocolate (9%) and alcohol (4%) (Supplementary Table 1).

During a median follow-up time of 19.2 years, we identified 1142 cases of overall breast cancer. There were 136 cases of pre- and 975 cases of post-menopausal breast cancer during median follow-up times of 11.9 and 15.8 years, respectively. Dietary NEAC was not associated with the hazard of breast cancer in the age-adjusted analysis (*p*-value for trend = 0.330), but a trend was observed after multivariable adjustment (highest vs lowest quartile, HR = 0.85, 95% CI 0.69-1.04, *p*-value for trend = 0.048). However, the Wald test was not statistically significant (*p*-value = 0.138) and regarding the direction of the associations the observed trend was not monotonic (HRs of 1.07 (second category), 0.95 (third category) and 0.85 (fourth category)). NEAC was not associated with pre-menopausal breast cancer in either the age-adjusted (*p*-value for trend = 0.619) or the multivariable-adjusted model (*p*-value for trend = 0.172). On the other hand, women in the highest quartile of NEAC intake had a 24% decreased hazard of post-menopausal breast cancer after multivariable adjustment (HR = 0.76, 95% CI 0.60-0.96 for women with NEAC≥11.8 compared to NEAC<6.7, *p*-value for trend = 0.010, e-value = 1.960) (Tables 2 and 3). When the logarithmic transformation of dietary NEAC was included as a continuous variable, the trend patterns remained consistent (breast cancer: HR = 0.85 (95% CI = 0.71;1.01), p-value = 0.064; pre-menopausal breast cancer: HR = 1.62 (95% CI = 0.54; 4.85), *p*-value = 0.387; post-menopausal breast cancer: HR = 0.80 (95% CI = 0.66; 0.97), *p*-value = 0.024). In addition, the spline analysis revealed no departures from linearity (*p*-value for non-linearity: breast cancer p-value = 0.650, pre-menopausal breast cancer p-value = 0.866, post-menopausal breast cancer p-value = 0.592) (Supplementary Fig. 2). In the stratified analyses, we did not find any difference in the associations for pre-menopausal breast cancer (Table 4). For post-menopausal breast cancer we found the strongest evidence for an inverse association between NEAC and post-menopausal breast cancer in women who did not smoke (HR = 0.78, 95% CI 0.61-0.99, comparing women with the highest NEAC intake, ≥11.8, to women with the lowest intake, NEAC<6.7, *p*-value for trend = 0.057, *p*-value for Wald test = 0.035) and in women with normal BMI (HR = 0.69 for highest vs lowest NEAC intake, 95% CI 0.50-0.94, *p*-value for trend = 0.021, *p*-value for Wald test = 0.078)) (Table 5).

**Table 2:** Age-adjusted hazard ratios of breast cancer for dietary NEAC intake in the National March Cohort

Outcome	Dietary NEAC^a^				*P*_trend_	*P*_Wald_
Breast cancer						
NEAC	<6.7	6.7- 8.9	8.9-11.7	≥11.7		
No. of cases	278	306	284	274		
Person-years	108,963	108,676	108,779	108,117		
HR (95%CI)	1.00 (ref)	1.08 (0.92-1.27)	1.00 (0.85-1.18)	0.95 (0.80-1.12)	0.330	0.486
Premenopausal breast cancer						
NEAC	<6.6	6.6-8.8	8.8-11.6	≥11.6		
No. of cases	35	33	30	38		
Person-years	31,986	31,870	31,724	30,897		
HR (95%CI)	1.00 (ref)	0.97 (0.61-1.57)	0.89 (0.55-1.45)	1.12 (0.71-1.78)	0.619	0.818
Postmenopausal breast cancer						
NEAC	<6.7	6.7-8.9	8.9-11.8	≥11.8		
No. of cases	234	261	256	224		
Person-years	71,689	73,466	73,675	74,203		
HR (95%CI)	1.00 (ref)	1.08 (0.90-1.28)	1.05 (0.88-1.26)	0.91 (0.76-1.10)	0.216	0.283

*Abbreviations*: *CI* Confidence interval, *HR* Hazard ratio

^a^NEAC was measured with the NEAC assay expressed in mmol Fe2+ equivalents/day

**Table 3:** Fully-adjusted hazard ratios of breast cancer for dietary NEAC intake in the National March Cohort

Outcome	Dietary NEAC^a^				*P*_trend_	*P*_Wald_
Breast Cancer^b^						
NEAC	<6.7	6.7- 8.9	8.9-11.7	≥11.7		
No. of cases	205	231	212	195		
Person-years	79,955	81,828	83,516	82,215		
HR (95%CI)	1.00 (ref)	1.07 (0.88-1.29)	0.95 (0.78-1.15)	0.85 (0.69-1.04)	0.048	0.138
Premenopausal Breast Cancer^c^						
NEAC	<6.6	6.6-8.8	8.8-11.6	≥11.6		
No. of cases	25	26	24	35		
Person-years	26,291	26,697	26,721	26,045		
HR (95%CI)	1.00 (ref)	1.07 (0.62-1.86)	0.99 (0.56-1.75)	1.44 (0.84-2.47)	0.172	0.438
Postmenopausal Breast Cancer^d^						
NEAC	<6.7	6.7-8.9	8.9-11.8	≥11.8		
No. of cases	173	194	194	153		
Person-years	50,024	52,931	54,378	54,333		
HR (95%CI)	1.00 (ref)	1.04 (0.84-1.27)	1.00 (0.81-1.23)	0.76 (0.60-0.96)	0.010	0.029

*Abbreviations*: *CI* Confidence interval, *HR* Hazard ratio

^a^NEAC was measured with the NEAC assay expressed in mmol Fe2+ equivalents/day

^b^Hazard ratios from fully-adjusted models for breast cancer overall are adjusted for: age, body mass index, menopausal status, energy intake, educational level, cigarette smoking status, alcohol drinking, coffee drinking, physical activity, vitamins and minerals use, contraceptive pill use, hormone replacement therapy, age at the first menstruation, number of children and childlessness. Participants with missing values in covariates are excluded from the models

^c^Hazard ratios from fully-adjusted models for premenopausal breast cancer are adjusted for: age, body mass index, energy intake, educational level, cigarette smoking status, alcohol drinking, coffee drinking, physical activity, vitamins and minerals use, contraceptive pill use, age at the first menstruation, number of children and childlessness. Participants with missing values in covariates are excluded from the models

^d^Hazard ratios from fully-adjusted models for postmenopausal breast cancer are adjusted for: age, body mass index, energy intake, educational level, cigarette smoking status, alcohol drinking, coffee drinking, physical activity, vitamins and minerals use, contraceptive pill use, hormone replacement therapy, age at the first menstruation, number of children and childlessness. Participants with missing values in covariates are excluded from the models

**Table 4:** Hazard ratios of premenopausal breast cancer for dietary NEAC intake by subgroups in the National March Cohort

Stratification variable	Dietary NEAC^a^				*P*_trend_	*P*_Wald_
	<6.6	6.6-8.8	8.8-11.6	≥11.6		
Vitamins and minerals use						
* No*						
No. of cases	15	15	13	18		
Person-years	18,117	17,403	16,717	16,520		
HR (95%CI)	1.00 (ref)	1.12 (0.54-2.03)	0.95 (0.45-2.02)	1.55 (0.75-3.19)	0.263	0.544
* Yes*						
No. of cases	10	11	11	17		
Person-years	8174	9294	10,005	9525		
HR (95%CI)^b^	1.00 (ref)	1.03 (0.43-2.46)	0.99 (0.41-2.38)	1.42 (0.63-3.21)	0.346	0.746
Cigarette smoking						
* Former or never*						
No. of cases	20	22	21	32		
Person-years	22,702	23,985	24,226	24,270		
HR (95%CI)^b^	1.00 (ref)	1.07 (0.58-1.97)	1.00 (0.54-1.86)	1.45 (0.81-2.61)	0.182	0.480
* Current*						
No. of cases	5	4	3	3		
Person-years	3589	2713	2495	1775		
HR (95%CI)^b^	1.00 (ref)	1.15 (0.29-4.46)	0,92 (0.21-4.06)	1.41 (0.30-6.72)	0.752	0.963
Body mass index						
* Normal*						
No. of cases	15	21	19	28		
Person-years	19,016	20,283	20,186	19,960		
HR (95%CI)^b^	1.00 (ref)	1.43 (0.73; 2.78)	1.28 (0.64; 2.55)	1.84 (0.96; 3.53)	0.083	0.305
* Overweight or Obese*						
No. of cases	10	5	5	7		
Person-years	7275	6414	6535	6086		
HR (95%CI)^b^	1.00 (ref)	0.50 (0.17; 1.48)	0.55 (0.18; 1.64)	0.81 (0.28; 2.29)	0.788	0.546

*Abbreviations*: *CI* Confidence interval, *HR* Hazard ratio

^a^NEAC was measured with the NEAC assay expressed in mmol Fe2+ equivalents/day

^b^Fully-adjusted hazard ratios for premenopausal breast cancer are adjusted by: body mass index energy intake, educational level, cigarette smoking status, alcohol drinking, coffee drinking, physical activity, vitamins and minerals use, contraceptive pill use, age at the first menstruation, number of children and childlessness. Participants with missing values in covariates are excluded from the models

**Table 5:** Hazard ratios of postmenopausal breast cancer for dietary NEAC intake by subgroups in the National March Cohort

Stratification variable	Dietary NEAC^a^				*P*_trend_	*P*_Wald_
	<6.7	6.7-8.9	8.9-11.8	≥11.8		
Hormone replacement therapy						
* No*						
No. of cases	103	98	111	82		
Person-years	33,887	34,338	34,942	33,091		
HR (95%CI)^b^	1.00 (ref)	0.92 (0.69-1.21)	1.03 (0.78-1.35)	0.80 (0.58-1.08)	0.217	0.336
* Yes*						
No. of cases	70	96	83	71		
Person-years	16,136	18,593	19,437	21,242		
HR (95%CI)^b^	1.00 (ref)	1.19 (0.87-1.63)	0.98 (0.71-1.36)	0.72 (0.51-1.03)	0.015	0.025
Vitamins and minerals use						
* No*						
No. of cases	105	129	127	98		
Person-years	32, 643	34,625	34,738	35,552		
HR (95%CI)^b^	1.00 (ref)	1.12 (0.86-1.45)	1.08 (0.83-1.41)	0.76 (0.57-1.02)	0.030	0.029
* Yes*						
No. of cases	68	65	67	55		
Person-years	17,380	18,306	19,641	18,780		
HR (95%CI)^b^	1.00 (ref)	0.89 (0.63-1.26)	0.88 (0.62-1.25)	0.74 (0.51-1.09)	0.138	0.503
Cigarette smoking						
* Former or never*						
No. of cases	147	176	181	144		
Person-years	44,243	48,687	50,600	51,494		
HR (95%CI)^b^	1.00 (ref)	1.07 (0.85-1.33)	1.04 (0.83-1.30)	0.78 (0.61-0.99)	0.057	0.035
* Current*						
No. of cases	26	18	13	9		
Person-years	5781	4244	3778	2839		
HR (95%CI)^b^	1.00 (ref)	0.88 (0.48-1.64)	0.72 (0.36-1.45)	0.57 (0.25-1.28)	0.139	0.533
Body mass index						
* Normal*						
No. of cases	95	94	113	89		
Person-years	27,902	30,451	34,046	35,504		
HR (95%CI)^b^	1.00 (ref)	0.89 (0.67-1.19)	0.95 (0.71-1.25)	0.69 (0.50-0.94)	0.021	0.078
* Overweight or Obese*						
No. of cases	78	100	81	64		
Person-years	22,121	22,479	20,333	18,829		
HR (95%CI)^b^	1.00 (ref)	1.20 (0.89-1.62)	1.05 (0.76-1.44)	0.86 (0.61-1.22)	0.223	0.243

*Abbreviations*: *CI* Confidence interval, *HR* Hazard ratio

^a^NEAC was measured with the NEAC assay expressed in mmol Fe2+ equivalents/day

^b^Fully-adjusted hazard ratios for postmenopausal breast cancer are adjusted by: body mass index energy intake, educational level, cigarette smoking status, alcohol drinking, coffee drinking, physical activity, vitamins and minerals use, contraceptive pill use, hormone replacement therapy, age at the first menstruation, number of children and childlessness. Participants with missing values in covariates are excluded from the models

Analyses on food-source-specific NEAC revealed that women with the highest intake of NEAC from fruits and vegetables had a 21% lower hazard of any breast cancer compared to the group with the lowest intake (HR = 0.79, 95% CI 0.64-0.97, *p*-value for trend = 0.019, *p*-value for Wald test = 0.113). In the pre-menopausal subsample, age-adjusted models revealed a statistically significant 42% decrease in the hazard of breast cancer when comparing groups with the highest and the lowest NEAC intake from fruits and vegetables (HR = 0.58, 95% CI 0.36-0.95), but *p*-values for trend (*p* = 0.057) and for Wald test (*p* = 0.143) were not statistically significant and the association was weaker in the fully-adjusted model, showing a 38% hazard decrease (HR = 0.62, 95% CI 0.36-1.08, p-value for trend = 0.107, *p*-value for Wald test = 0.378). On the other hand, women in the highest quartile of NEAC intake from fruits and vegetables had a lower hazard of post-menopausal breast cancer after multivariable adjustment (HR = 0.81, 95% CI 0.65-1.02, *p*-value for trend = 0.027, *p*-value for Wald test = 0.098) (Table 6). NEAC from grains was not associated with a lower risk of developing breast cancer (Table 7). Analyses on NEAC from tea consumption revealed that women with the highest intake of NEAC from tea had a 99% higher hazard of premenopausal breast cancer compared to non-consumers (HR = 1.99, 95% CI 1.19-3.33, *p*-value for trend = 0.010) (Supplementary Table 2).

**Table 6:** Hazard ratios of breast cancer for dietary NEAC intake from fruits and vegetables in the National March Cohort

Outcome	Dietary NEAC^a^ from Fruits and Vegetables				*P*_trend_	*P*_Wald_
Breast cancer						
NEAC	<1.3	1.3- 2.0	2.0-2.9	≥2.9		
Age-adjusted models						
No. of cases	274	281	293	289		
Person-years	108,912	108,743	107,845	106,734		
HR (95%CI)	1.00 (ref)	0.91 (0.77-1.08)	0.91 (0.77-1.07)	0.86 (0.73-1.02)	0.110	0.370
Fully-adjusted models^b^						
No. of cases	197	218	229	195		
Person-years	82,270	82,381	82,286	79,409		
HR (95%CI)	1.00 (ref)	0.95 (0.78-1.15)	0.93 (0.77-1.13)	0.79 (0.64-0.97)	0.019	0.113
Premenopausal breast cancer						
NEAC	<1.2	1.2-1.8	1.8-2.6	≥2.6		
Age-adjusted models						
No. of cases	45	31	35	25		
Person-years	36,128	31,720	29,694	28,274		
HR (95%CI)	1.00 (ref)	0.68 (0.43-1.08)	0.79 (0.51-1.23)	0.58 (0.36-0.95)	0.057	0.143
Fully-adjusted models^c^						
No. of cases	35	27	27	21		
Person-years	30,278	26,323	25,164	23,516		
HR (95%CI)	1.00 (ref)	0.77 (0.46-1.27)	0.76 (0.46-1.27)	0.62 (0.36-1.08)	0.107	0.378
Postmenopausal Breast Cancer						
NEAC	<1.4	1.4-2.1	2.1-2.9	≥2.9		
Age-adjusted models						
No. of cases	236	247	243	244		
Person-years	68,162	72,377	73,966	76,999		
HR (95%CI)	1.00 (ref)	0.98 (0.82-1.17)	0.93 (0.78-1.12)	0.89 (0.74-1.06)	0.168	0.590
Fully-adjusted models^d^						
No. of cases	165	196	187	162		
Person-years	48,094	52,821	54,541	55,553		
HR (95%CI)	1.00 (ref)	1.06 (0.86-1.30)	0.96 (0.78-1.19)	0.81 (0.65-1.02)	0.027	0.098

*Abbreviations*: *CI* Confidence interval, *HR* Hazard ratio

^a^NEAC was measured with the NEAC assay expressed in mmol Fe2+ equivalents/day

^b^Hazard ratios from fully-adjusted models for breast cancer overall are adjusted for: age, body mass index, menopausal status, energy intake, educational level, cigarette smoking status, alcohol drinking, coffee drinking, physical activity, vitamins and minerals use, contraceptive pill use, hormone replacement therapy, age at the first menstruation, number of children and childlessness. Participants with missing values in covariates are excluded from the models

^c^Hazard ratios from fully-adjusted models for premenopausal breast cancer are adjusted for: age, body mass index, energy intake, educational level, cigarette smoking status, alcohol drinking, coffee drinking, physical activity, vitamins and minerals use, contraceptive pill use, age at the first menstruation, number of children and childlessness. Participants with missing values in covariates are excluded from the models

^d^Hazard ratios from fully-adjusted models for postmenopausal breast cancer are adjusted for: age, body mass index, energy intake, educational level, cigarette smoking status, alcohol drinking, coffee drinking, physical activity, vitamins and minerals use, contraceptive pill use, hormone replacement therapy, age at the first menstruation, number of children and childlessness. Participants with missing values in covariates are excluded from the models

**Table 7:** Hazard ratios of breast cancer for dietary NEAC intake from grains in the National March Cohort

Outcome	Dietary NEAC^a^ from Grains				*P*_trend_	*P*_Wald_
Breast cancer						
NEAC	<1.2	1.2-1.5	1.5-2.0	≥2.0		
Age-adjusted models						
No. of cases	251	301	280	303		
Person-years	109,577	108,637	108,538	105,295		
HR (95%CI)	1.00 (ref)	1.16 (0.98-1.37)	1.02 (0.86-1.21)	1.04 (0.87-1.23)	0.825	0.301
Fully-adjusted models^b^						
No. of cases	189	211	216	222		
Person-years	81,948	84,278	83,585	76,249		
HR (95%CI)	1.00 (ref)	1.03 (0.85-1.25)	1.01 (0.83-1.23)	1.05 (0.86-1.29)	0.630	0.953
Premenopausal breast cancer						
NEAC	<1.1	1.1-1.4	1.4-1.8	≥1.8		
Age-adjusted models						
No. of cases	34	34	36	32		
Person-years	32,762	32,022	30,931	30,173		
HR (95%CI)	1.00 (ref)	0.99 (0.62-1.59)	1.05 (0.66-1.67)	0.95 (0.59-1.54)	0.864	0.984
Fully-adjusted models^c^						
No. of cases	28	28	28	26		
Person-years	26,734	27,030	26,255	25,258		
HR (95%CI)	1.00 (ref)	0.93 (0.55-1.57)	0.93 (0.55-1.58)	0.88 (0.51-1.51)	0.654	0.973
Postmenopausal breast cancer						
NEAC	<1.2	1.2-1.5	1.5-2.1	≥2.1		
Age-adjusted models						
No. of cases	207	267	236	258		
Person-years	70,046	71,073	73,217	76,914		
HR (95%CI)	1.00 (ref)	1.26 (1.05-1.51)	1.07 (0.88-1.28)	1.08 (0.90-1.30)	0.949	0.071
Fully-adjusted models^d^						
No. of cases	153	186	179	191		
Person-years	49,866	53,122	54,061	53,688		
HR (95%CI)	1.00 (ref)	1.14 (0.92-1.41)	1.06 (0.86-1.32)	1.15 (0.93-1.43)	0.316	0.545

*Abbreviations*: *CI* Confidence interval, *HR* Hazard ratio

^a^NEAC was measured with the NEAC assay expressed in mmol Fe2+ equivalents/day

^b^Hazard ratios from fully-adjusted models for breast cancer overall are adjusted for: age, body mass index, menopausal status, energy intake, educational level, cigarette smoking status, alcohol drinking, coffee drinking, physical activity, vitamins and minerals use, contraceptive pill use, hormone replacement therapy, age at the first menstruation, number of children and childlessness. Participants with missing values in covariates are excluded from the models

^c^Hazard ratios from fully-adjusted models for premenopausal breast cancer are adjusted for: age, body mass index, energy intake, educational level, cigarette smoking status, alcohol drinking, coffee drinking, physical activity, vitamins and minerals use, contraceptive pill use, age at the first menstruation, number of children and childlessness. Participants with missing values in covariates are excluded from the models

^d^Hazard ratios from fully-adjusted models for postmenopausal breast cancer are adjusted for: age, body mass index, energy intake, educational level, cigarette smoking status, alcohol drinking, coffee drinking, physical activity, vitamins and minerals use, contraceptive pill use, hormone replacement therapy, age at the first menstruation, number of children and childlessness. Participants with missing values in covariates are excluded from the models

### Sensitivity analyses

The proportion of missing values varied between 0.2% for alcohol consumption and 9.6% for physical activity (Table 1). The analysis on imputed data included 6349 additional women for the multivariable-adjusted model and substantially confirmed the results of the complete-case analysis in terms of direction of the association, even if statistical significance was not demonstrated (breast cancer: highest vs lowest NEAC quartile, HR = 0.89, 95% CI 0.74-1.08, *p*-value for trend = 0.091; post-menopausal breast cancer: HR = 0.84 (0.69-1.03), p-value for trend = 0.057) (Supplementary Tables 3-5). The adjusted HR of developing breast cancer in post-menopausal women for the highest vs. lowest category of NEAC, was 0.84 (95% CI 0.69-1.03, *p*-value for trend = 0.057) (Supplementary Table 3) and the strongest association was found in post-menopausal women who did not use vitamin and mineral supplements (HR = 0.80, 95% CI 0.62-1.04; *p*-value for trend = 0.039). Individuals added in this sensitivity analysis less frequently had a normal BMI compared to those included in the main analysis (59.58% vs 62.01%, *p*-value = 0.003) while no differences were observed by smoking habits. Exclusion of the first 2 years of follow-up confirmed the results of the main analysis (Supplementary Table 6-9) and showed a 26% decreased hazard of post-menopausal breast cancer in the group with the highest intake of NEAC compared to the group with the lowest intake (adjusted HR = 0.74, 95% CI 0.58-0.95, *p*-value for trend = 0.010) (Supplementary Table 6). Finally, the inverse association between NEAC intake and hazard of post-menopausal breast cancer was confirmed when follow-up was restricted to 10 years (highest vs. lowest category of NEAC: HR = 0.71 (95% CI 0.52-0.96, *p*-value for trend = 0.012) (Supplementary Table 10) as well as when menopausal age was imputed based on patients characteristics (highest vs. lowest category of NEAC: HR = 0.75 (95% CI 0.59-0.94, *p*-value for trend = 0.011) (Supplementary Table 11).

## Discussion

In this prospective cohort study, NEAC intake was inversely associated with the hazard of any breast cancer, and specifically with post-menopausal breast cancer. In particular, a high intake of NEAC from fruits and vegetables, was associated with a reduced risk of breast cancer. However, the evidence of an association between NEAC and breast cancer risk was weaker in the sensitivity analysis where missing values in covariates were imputed using multiple imputation, potentially due to the different distribution of some effect modifiers between the sample of individuals added after imputation and those already in the main analysis, as seen for BMI.

Our results corroborate and complement the finding of one previous study that found high NEAC intake, estimated through the FRAP assay, to be associated with a lower risk of post-menopausal breast cancer [15]. As the sample size for studying pre-menopausal breast cancer was limited, our results suggest that the inverse association between NEAC and breast cancer overall might be driven by the inverse association found for post-menopausal breast cancer.

Our results are consistent with those of the Sister cohort study conducted on 43,563 women, that found an antioxidant rich diet score to be inversely associated with the risk of post-menopausal breast cancer and positively associated with pre-menopausal breast cancer [33]. Our results suggest that the overall intake of antioxidants from dietary sources, particularly from fruits and vegetables, might provide an opportunity for breast cancer prevention, highlighting the importance of following a varied diet containing antioxidant ingredients from different foods. By assessing the effect of total NEAC and NEAC from different food groups we contribute to the understanding of the etiological mechanisms that are underlying the observed inverse association between healthy diet and risk of breast cancer. On the other hand, an increased risk for premenopausal breast cancer was associated with NEAC from tea consumption. This result was consistent with the positive association found between black tea consumption (amount of cups/day of tea vs no consumption) and breast cancer in a prospective Swedish cohort including 61,433 women [34]. However, in that study, the association did not vary by menopausal status and several works reported that high vs low consumption of tea significantly lowered the risk of breast cancer [35]. The observed increased risk of breast cancer might be due to unknown mechanisms or other unmeasured confounding variables.

Oxidative stress may have a role in breast cancer initiation, promotion and progression [36]. Exposure to a pro-oxidant environment enables reactive oxygen species, ROS, to directly interact with cellular components, such as molecules, proteins and nucleic acids, by altering their structure and function. Irreversible changes in DNA and the accumulation of permanent changes in the genome are central for tumor initiation and progression, and increased levels of oxidative DNA lesions have been implicated in the etiology of various cancers [37]. The wide array of antioxidant ingredients, such as vitamins and flavonoids in fruits and vegetables, are also endowed with other functional activity such as anti-inflammatory properties, tightly linked with antioxidants, that might be extremely important for breast cancer prevention [38, 39]. Moreover, flavonoids, which are a subclass of plant polyphenols, have anti-carcinogenic properties and are discussed as promising treatment options for the prevention and treatment of breast cancer. Flavonoids can directly interact with proteins, and thus modulate enzymes, transcription factors and cell surface receptors. Particularly interesting is the ability of flavonoids to modulate the tumor-associated macrophage function and components of the tumor microenvironment, however clear evidence in vivo is lacking [40].

Another important aspect is the postprandial increase of free radicals and inflammatory molecules caused through the ingestion of high energy, fat and sugar meals, which is one of the mechanisms leading to chronic oxidative and inflammatory stress in humans. It has been shown that the daily ingestion of antioxidant-rich foods can hamper the oxidative and inflammatory stress, providing better protection against free radical insults [38]. The dietary prevention of acute oxidative and inflammatory postprandial stress through the diet might be extremely important in the view of optimizing the antioxidant action of specific ingredients, such as flavonoids, characterized by a low bioavailability in body fluids. In a condition where the preventive action takes place during digestive processes after ingestion, the antioxidant concentration of flavonoids is much higher than in body fluids, before the metabolization processes start. In general, dietary antioxidants could act through an indirect action of reduction of postprandial stress and a direct reduction of free radicals in body fluids, reducing the risk of developing systemic oxidative and inflammatory stress, risk factors for breast cancer development [41, 42].

Several limitations of our study need to be discussed. First, although we used a validated food frequency questionnaire, we had made slight changes to the questionnaire, which might have changed its validity. Furthermore, dietary intake was only assessed once at baseline, which limited our ability to capture changes over time. Moreover, food questionnaires are known to suffer from many biases including measurement errors. Indeed, the content of bioactive ingredients depends on several factors that cannot be considered, such as the different species of food items or quality of the soil. Given the long follow-up period, participants might have changed their diet to some extent over their lifespan, thus the strength of any causal association is likely to become underestimated. However, considering the long latency period from exposure to breast cancer occurrence, the baseline exposure might play a more important role than recent changes. To account for this limitation, we also conducted a sensitivity analysis restricting follow-up to 10 years. Second, dietary intake was self-reported, which might lead to misclassification of our exposure variable. However, given the prospective design of the study the misclassification is likely to be non-differential [43], but might not be independent from the measurement error of other variables. Thus, the direction of the influence of this bias is not certain. In addition, NEAC values were only available for 66 out of 80 food items. However, the NEAC content for the missing items (meat, dairy products, sweets and pastries) is generally low and therefore the misclassification of exposure due to missing NEAC values is thought to be minimal. Moreover, as in any observational study, we cannot rule out the presence of residual confounding from unmeasured variables including diet quality indicators or other lifestyle behaviors. However, the e-value corresponding to the main association provided evidence of robustness of our findings to residual confounding. Additionally, menopausal age was self-reported, but the date of menopause was inferred for approximately half of the sample due to pre-menopausal status at time of the questionnaire or due to conflicting data, which could potentially result in inaccurate age at menopause. However, in the sensitivity analysis where age at menopause was imputed according to the characteristics of the women, the findings were confirmed. Third, selection bias might occur due to differential possibility of loss to follow-up across different exposure groups. However, the high completeness of Swedish Cancer Register and other registers allayed such a concern. Fourth, this study was conducted in the Swedish population. The results cannot be directly generalized to other populations. As in any study among humans– randomized or observational - external validity cannot be directly assessed but requires confirmation in other independent investigations. In general, however, causal associations tend to be consistently documented in different populations. Furthermore, the wide representation of participants from all over Sweden in our cohort provides reassurance that our findings are not only relevant for a selected group. Fifth, we could not investigate variation of the associations by breast cancer hormonal receptor status, because we lacked this information for the cancer patients. Furthermore, by measuring NEAC intake from food-frequency questionnaires, we cannot infer our results to total antioxidant capacity in plasma, because enzymatic activity is a major antioxidant defense [44, 45]. Finally, we conducted several subgroup analyses (pre- and post-menopausal), and we stratified for a number of factors (e.g., by cigarette smoking, vitamins and minerals use, hormone replacement therapy). Thus, some subsamples counted relatively small numbers of cases, which may limit power and interpretability of results.

Despite this limitation, the NEAC component of total antioxidant capacity is important because of the potential of the diet to directly modify NEAC. Finally, pre-menopausal breast cancer was less common than post-menopausal breast cancer in the SNMC, therefore the stratified analyses by potential effect modifiers may be more accurate for post-menopausal breast cancer.

Strengths of our study are the prospective design, the large size of the cohort and essentially complete follow-up through record linkages to well-managed registers. In addition, the SNMC participants were recruited during a nationwide fund-raising campaign for cancer research and were probably motivated to provide valid high-quality exposure information.

We conclude that dietary antioxidant intake from a diverse intake of plant foods, mainly fruits and vegetables, may have a protective effect on breast cancer risk, especially post-menopausal breast cancer, but causal effects of antioxidant intake, especially from fruits and vegetables, need to be better understood. While the majority of sensitivity analyses were consistent with our main findings, results were not fully confirmed after imputation of covariates. Future research is thus necessary to confirm our findings in order to improve our current knowledge on lifestyle and postmenopausal health.

## Supplementary Information

Supplementary Material 1.

Supplementary Material 2.

## Data Availability

The datasets analyzed during the current study are not publicly available due to ethical restrictions, but are available from the authors on reasonable request.

## Abbreviations

NEAC: Non-Enzymatic Antioxidant Capacity
FRAP: Ferric-Reducing Antioxidant Potential
TRAP: Total Radical-Trapping Antioxidant Parameter
TEAC: Plasma Trolox Equivalent Antioxidant Capacity
SNMC: Swedish National March Cohort
FFQ: Food frequency questionnaire
MET: Metabolic equivalent of task
BMI: Body mass index
HR: Hazard ratio
CI: Confidence interval
IQR: Interquartile range
DAG: Directed Acyclic Graph
