# Supplementary material for: Dietary non-enzymatic antioxidant capacity and risk of breast cancer: the Swedish National March Cohort
Source: BMC Cancer. 2025 Aug 13;25:1310. doi: 10.1186/s12885-025-14658-z (PMC12344984; doi:10.1186/s12885-025-14658-z)
Supplement: Supplementary file 1 — Supplementary Material 1. [file 12885_2025_14658_MOESM1_ESM.docx]

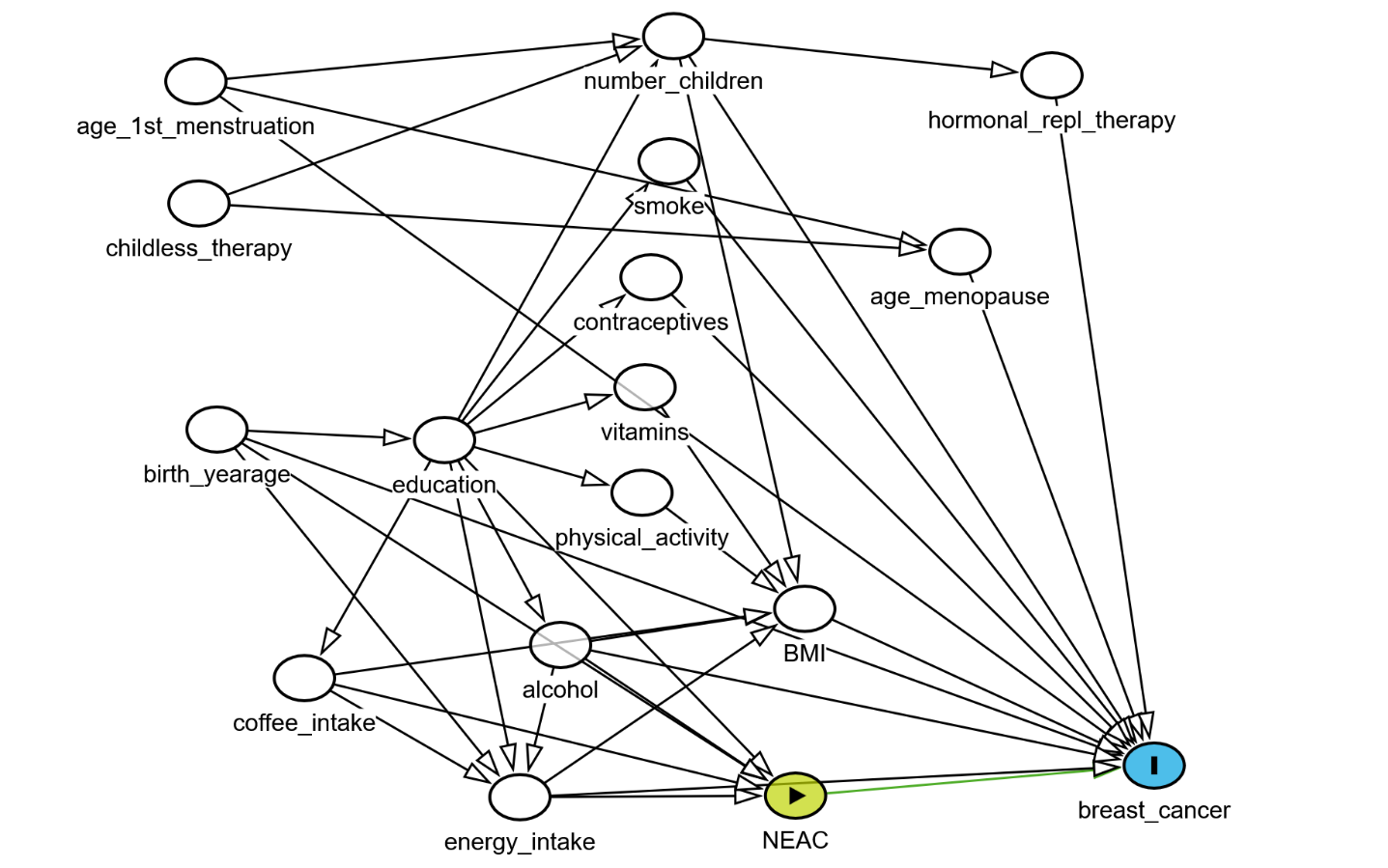


Supplementary Figure 1. Directed Acyclic Graph (DAG)

A

B


C

Supplementary Figure 2. Restricted cubic splines for modelling the association between dietary NEAC and breast cancer risk (4 knots at the 5th, 35th, 65th and 95th percentile): A) total breast cancer; B) premenopausal breast cancer; C) postmenopausal breast cancer.
