# Supplementary material for: Dietary non-enzymatic antioxidant capacity and risk of breast cancer: the Swedish National March Cohort
Source: BMC Cancer. 2025 Aug 13;25:1310. doi: 10.1186/s12885-025-14658-z (PMC12344984; doi:10.1186/s12885-025-14658-z)
Supplement: Supplementary file 2 — Supplementary Material 2. [file 12885_2025_14658_MOESM2_ESM.docx]

**Supplementary material**

**Supplementary Table 1. Main contributions of food groups to dietary NEAC.**

|  | **Overall**  **sample** | **Pre-menopausal**  **subsample** | **Post-menopausal**  **subsample** |
| --- | --- | --- | --- |
| **Tea** | 28% | 32% | 28% |
| **Fruit and vegetables** | 23% | 22% | 24% |
| **Grain** | 19% | 17% | 19% |
| **Chocolate** | 9% | 10% | 9% |
| **Alcohol** | 4% | 3% | 4% |
| **Other** | 17% | 16% | 16% |

**Supplementary Table 2.** **Hazard ratios of breast cancer for dietary NEAC intake from tea in the National March Cohort.**

| Outcome | Dietary NEAC ^a^ from Tea | | | | *P* _trend_ | *P* _Wald_ |
| --- | --- | --- | --- | --- | --- | --- |
| **Breast Cancer** |  |  |  |  |  |  |
| NEAC | 0 | 2.8-3.0 | 3.0-5.9 | ≥5.9 |  |  |
| **Age-adjusted models** |  |  |  |  |  |  |
| No. of cases | 540 | 206 | 199 | 197 |  |  |
| Person-years | 206,993 | 75,714 | 75,701 | 76,127 |  |  |
| HR (95%CI) | 1.00 (ref) | 1.14 (0.97-1.34) | 1.08 (0.92-1.27) | 1.05 (0.89-1.23) | 0.484 | 0.430 |
| **Fully-adjusted models** ^b^ |  |  |  |  |  |  |
| No. of cases | 394 | 156 | 148 | 145 |  |  |
| Person-years | 151,634 | 59,226 | 58,546 | 58,108 |  |  |
| HR (95%CI) | 1.00 (ref) | 1.14 (0.93-1.39) | 1.03 (0.85-1.26) | 1.00 (0.82-1.23) | 0.905 | 0.628 |
| **Premenopausal Breast Cancer** |  |  |  |  |  |  |
| NEAC | 0 | 2.8-3.0 | 3.0-5.9 | ≥5.9 |  |  |
| **Age-adjusted models** |  |  |  |  |  |  |
| No. of cases | 50 | 24 | 29 | 33 |  |  |
| Person-years | 53,613 | 24,721 | 24,608 | 23,534 |  |  |
| HR (95%CI) | 1.00 (ref) | 1.14 (0.70-1.85) | 1.39 (0.88-2.20) | 1.54 (0.99-2.40) | 0.052 | 0.218 |
| **Fully-adjusted models** ^c^ |  |  |  |  |  |  |
| No. of cases | 37 | 18 | 25 | 30 |  |  |
| Person-years | 44,316 | 20,783 | 20,989 | 19,668 |  |  |
| HR (95%CI) | 1.00 (ref) | 1.13 (0.62-2.05) | 1.71 (1.00-2.90) | 1.99 (1.19-3.33) | 0.010 | 0.040 |
| **Postmenopausal Breast Cancer** |  |  |  |  |  |  |
| NEAC | 0 | 2.8-3.0 | 3.0-5.9 | ≥5.9 |  |  |
| **Age-adjusted models** |  |  |  |  |  |  |
| No. of cases | 474 | 178 | 166 | 157 |  |  |
| Person-years | 146,287 | 48,363 | 49,310 | 49,074 |  |  |
| HR (95%CI) | 1.00 (ref) | 1.15 (0.97-1.37) | 1.04 (0.87-1.25) | 1.00 (0.83-1.19) | 0.895 | 0.432 |
| **Fully-adjusted models** ^d^ |  |  |  |  |  |  |
| No. of cases | 343 | 137 | 122 | 112 |  |  |
| Person-years | 102,706 | 36,592 | 36,458 | 35,910 |  |  |
| HR (95%CI) | 1.00 (ref) | 1.17 (0.94-1.44) | 0.99 (0.80-1.23) | 0.92 (0.73-1.15) | 0.556 | 0.357 |

Abbreviations: CI=confidence interval, HR=hazard ratio.

^a^ NEAC was measured with the NEAC assay expressed in mmol Fe2+ equivalents/day.

^b^ Hazard ratios from fully-adjusted models for breast cancer overall are adjusted for: age, body mass index, menopausal status, energy intake, educational level, cigarette smoking status, alcohol drinking, coffee drinking, physical activity, vitamins and minerals use, contraceptive pill use, hormone replacement therapy, age at the first menstruation, number of children and childlessness. Participants with missing values in covariates are excluded from the models.

^c^ Hazard ratios from fully-adjusted models for premenopausal breast cancer are adjusted for: age, body mass index, energy intake, educational level, cigarette smoking status, alcohol drinking, coffee drinking, physical activity, vitamins and minerals use, contraceptive pill use, age at the first menstruation, number of children and childlessness. Participants with missing values in covariates are excluded from the models.

^d^ Hazard ratios from fully-adjusted models for postmenopausal breast cancer are adjusted for: age, body mass index, energy intake, educational level, cigarette smoking status, alcohol drinking, coffee drinking, physical activity, vitamins and minerals use, contraceptive pill use, hormone replacement therapy, age at the first menstruation, number of children and childlessness. Participants with missing values in covariates are excluded from the models.

**Supplementary Table 3. Hazard ratios of breast cancer for dietary NEAC intake in the National March Cohort after imputation of missing values.**

| Outcome | Dietary NEAC | | | | *P* _trend_ | *P* _Wald_ |
| --- | --- | --- | --- | --- | --- | --- |
| **Breast Cancer** |  |  |  |  |  |  |
| NEAC | <6.7 | 6.7- 8.9 | 8.9-11.7 | ≥11.7 |  |  |
| No. of cases | 278 | 306 | 284 | 274 |  |  |
| Person-years | 108,963 | 108,676 | 108,779 | 108,117 |  |  |
| HR (95%CI) ^a^ | 1.00 (ref) | 1.08 (0.92-1.27) | 1.00 (0.85-1.18) | 0.95 (0.80-1.12) | 0.330 | 0.486 |
| HR (95%CI) ^b^ | 1.00 (ref) | 1.08 (0.91-1.28) | 1.00 (0.84-1.19) | 0.89 (0.74-1.08) | 0.091 | 0.218 |
| **Premenopausal Breast Cancer** |  |  |  |  |  |  |
| NEAC | <6.6 | 6.6-8.8 | 8.8-11.6 | ≥11.6 |  |  |
| No. of cases | 35 | 33 | 30 | 38 |  |  |
| Person-years | 31,986 | 31,870 | 31,724 | 30,897 |  |  |
| HR (95%CI) ^a^ | 1.00 (ref) | 0.97 (0.61-1.57) | 0.89 (0.55-1.45) | 1.12 (0.71-1.78) | 0.619 | 0.818 |
| HR (95%CI)  ^c^ | 1.00 (ref) | 0.98 (0.61-1.60) | 0.85 (0.51-1.40) | 1.11 (0.68-1.81) | 0.700 | 0.758 |
| **Postmenopausal Breast Cancer** |  |  |  |  |  |  |
| NEAC | <6.7 | 6.7-8.9 | 8.9-11.8 | ≥11.8 |  |  |
| No. of cases | 234 | 261 | 256 | 224 |  |  |
| Person-years | 71,689 | 73,466 | 73,675 | 74,203 |  |  |
| HR (95%CI) ^a^ | 1.00 (ref) | 1.08 (0.90-1.28) | 1.05 (0.88-1.26) | 0.91 (0.76-1.10) | 0.216 | 0.283 |
| HR (95%CI) ^d^ | 1.00 (ref) | 1.06 (0.88-1.27) | 1.05 (0.87-1.27) | 0.84 (0.69-1.03) | 0.057 | 0.076 |

Abbreviations: CI=confidence interval, HR=hazard ratio.

^a^ Age-adjusted hazard ratios.

^b^ Fully-adjusted hazard ratios for breast cancer overall are adjusted for: age, body mass index, menopausal status, energy intake, educational level, cigarette smoking status, alcohol drinking, coffee drinking, physical activity, vitamins and minerals use, contraceptive pill use, hormone replacement therapy, age at the first menstruation, number of children and childlessness.

^c^ Fully-adjusted hazard ratios for premenopausal breast cancer are adjusted for: age, body mass index, energy intake, educational level, cigarette smoking status, alcohol drinking, coffee drinking, physical activity, vitamins and minerals use, contraceptive pill use, age at the first menstruation, number of children and childlessness.

^d^ Fully-adjusted hazard ratios for postmenopausal breast cancer are adjusted for: age, body mass index energy intake, educational level, cigarette smoking status, alcohol drinking, coffee drinking, physical activity, vitamins and minerals use, contraceptive pill use, hormone replacement therapy, age at the first menstruation, number of children and childlessness.

**Supplementary Table 4. Hazard ratios of breast cancer for dietary NEAC intake from fruits and vegetables in the National March Cohort after imputation of missing values.**

| Outcome | Dietary NEAC from Fruits and Vegetables | | | | *P* _trend_ | *P* _Wald_ |
| --- | --- | --- | --- | --- | --- | --- |
| **Breast Cancer** |  |  |  |  |  |  |
| NEAC | <1.3 | 1.3- 2.0 | 2.0-2.9 | ≥2.9 |  |  |
| No. of cases | 274 | 281 | 293 | 289 |  |  |
| Person-years | 108,912 | 108,743 | 107,845 | 106,734 |  |  |
| HR (95%CI) ^a^ | 1.00 (ref) | 0.91 (0.77-1.08) | 0.91 (0.77-1.07) | 0.86 (0.73-1.02) | 0.110 | 0.370 |
| HR (95%CI) ^b^ | 1.00 (ref) | 0.90 (0.76-1.07) | 0.88 (0.74-1.05) | 0.83 (0.70-0.99) | 0.051 | 0.233 |
| **Premenopausal Breast Cancer** |  |  |  |  |  |  |
| NEAC | <1.2 | 1.2-1.8 | 1.8-2.6 | ≥2.6 |  |  |
| No. of cases | 45 | 31 | 35 | 25 |  |  |
| Person-years | 36,128 | 31,720 | 29,694 | 28,274 |  |  |
| HR (95%CI) ^a^ | 1.00 (ref) | 0.68 (0.43-1.08) | 0.79 (0.51-1.23) | 0.58 (0.36-0.95) | 0.057 | 0.143 |
| HR (95%CI)  ^c^ | 1.00 (ref) | 0.65 (0.41-1.03) | 0.70 (0.44-1.10) | 0.55 (0.33-0.90) | 0.031 | 0.082 |
| **Postmenopausal Breast Cancer** |  |  |  |  |  |  |
| NEAC | <1.4 | 1.4-2.1 | 2.1-2.9 | ≥2.9 |  |  |
| No. of cases | 236 | 247 | 243 | 244 |  |  |
| Person-years | 68,162 | 72,377 | 73,966 | 76,999 |  |  |
| HR (95%CI) ^a^ | 1.00 (ref) | 0.98 (0.82-1.17) | 0.93 (0.78-1.12) | 0.89 (0.74-1.06) | 0.168 | 0.590 |
| HR (95%CI) ^d^ | 1.00 (ref) | 0.95 (0.79-1.15) | 0.92 (0.76-1.10) | 0.87 (0.72-1.05) | 0.126 | 0.497 |

Abbreviations: CI=confidence interval, HR=hazard ratio.

^a^ Age-adjusted hazard ratios.

^b^ Fully-adjusted hazard ratios for breast cancer overall are adjusted for: age, body mass index, menopausal status, energy intake, educational level, cigarette smoking status, alcohol drinking, coffee drinking, physical activity, vitamins and minerals use, contraceptive pill use, hormone replacement therapy, age at the first menstruation, number of children and childlessness.

^c^ Fully-adjusted hazard ratios for premenopausal breast cancer are adjusted for: age, body mass index, energy intake, educational level, cigarette smoking status, alcohol drinking, coffee drinking, physical activity, vitamins and minerals use, contraceptive pill use, age at the first menstruation, number of children and childlessness.

^d^ Fully-adjusted hazard ratios for postmenopausal breast cancer are adjusted for: age, body mass index energy intake, educational level, cigarette smoking status, alcohol drinking, coffee drinking, physical activity, vitamins and minerals use, contraceptive pill use, hormone replacement therapy, age at the first menstruation, number of children and childlessness.

**Supplementary Table 5. Hazard ratios of breast cancer for dietary NEAC intake from grains in the National March Cohort after imputation of missing values.**

| Outcome | Dietary NEAC from Grains | | | | *P* _trend_ | *P* _Wald_ |
| --- | --- | --- | --- | --- | --- | --- |
| **Breast Cancer** |  |  |  |  |  |  |
| NEAC | <1.2 | 1.2-1.5 | 1.5-2.0 | ≥2.0 |  |  |
| No. of cases | 251 | 301 | 280 | 303 |  |  |
| Person-years | 109,577 | 108,637 | 108,538 | 105,295 |  |  |
| HR (95%CI) ^a^ | 1.00 (ref) | 1.16 (0.98-1.37) | 1.02 (0.86-1.21) | 1.04 (0.87-1.23) | 0.825 | 0.301 |
| HR (95%CI) ^b^ | 1.00 (ref) | 1.13 (0.95-1.35) | 1.03 (0.87-1.23) | 1.06 (0.89-1.27) | 0.814 | 0.516 |
| **Premenopausal Breast Cancer** |  |  |  |  |  |  |
| NEAC | <1.1 | 1.1-1.4 | 1.4-1.8 | ≥1.8 |  |  |
| No. of cases | 34 | 34 | 36 | 32 |  |  |
| Person-years | 32,762 | 32,022 | 30,931 | 30,173 |  |  |
| HR (95%CI) ^a^ | 1.00 (ref) | 0.99 (0.62-1.59) | 1.05 (0.66-1.67) | 0.95 (0.59-1.54) | 0.864 | 0.984 |
| HR (95%CI)  ^c^ | 1.00 (ref) | 0.94 (0.58-1.52) | 1.02 (0.63-1.64) | 0.89 (0.55-1.46) | 0.702 | 0.950 |
| **Postmenopausal Breast Cancer** |  |  |  |  |  |  |
| NEAC | <1.2 | 1.2-1.5 | 1.5-2.1 | ≥2.1 |  |  |
| No. of cases | 207 | 267 | 236 | 258 |  |  |
| Person-years | 70,046 | 71,073 | 73,217 | 76,914 |  |  |
| HR (95%CI) ^a^ | 1.00 (ref) | 1.26 (1.05-1.51) | 1.07 (0.88-1.28) | 1.08 (0.90-1.30) | 0.949 | 0.071 |
| HR (95%CI) ^d^ | 1.00 (ref) | 1.23 (1.02-1.48) | 1.07 (0.88-1.29) | 1.13 (0.94-1.37) | 0.540 | 0.161 |

Abbreviations: CI=confidence interval, HR=hazard ratio.

^a^ Age-adjusted hazard ratios.

^b^ Fully-adjusted hazard ratios for breast cancer overall are adjusted for: age, body mass index, menopausal status, energy intake, educational level, cigarette smoking status, alcohol drinking, coffee drinking, physical activity, vitamins and minerals use, contraceptive pill use, hormone replacement therapy, age at the first menstruation, number of children and childlessness.

^c^ Fully-adjusted hazard ratios for premenopausal breast cancer are adjusted for: age, body mass index, energy intake, educational level, cigarette smoking status, alcohol drinking, coffee drinking, physical activity, vitamins and minerals use, contraceptive pill use, age at the first menstruation, number of children and childlessness.

^d^ Fully-adjusted hazard ratios for postmenopausal breast cancer are adjusted for: age, body mass index energy intake, educational level, cigarette smoking status, alcohol drinking, coffee drinking, physical activity, vitamins and minerals use, contraceptive pill use, hormone replacement therapy, age at the first menstruation, number of children and childlessness.

**Supplementary Table 6. Hazard ratios of breast cancer for dietary NEAC intake in the National March Cohort after exclusion of the first two years of follow-up.**

| Outcome | Dietary NEAC | | | | *P* _trend_ | *P* _Wald_ |
| --- | --- | --- | --- | --- | --- | --- |
| **Breast Cancer** |  |  |  |  |  |  |
| NEAC | <6.7 | 6.7- 8.9 | 8.9-11.7 | ≥11.7 |  |  |
| **Age-adjusted models** |  |  |  |  |  |  |
| No. of cases | 251 | 273 | 260 | 248 |  |  |
| Person-years | 96,586 | 96,291 | 96,400 | 95,751 |  |  |
| HR (95%CI) | 1.00 (ref) | 1.07 (0.90-1.27) | 1.02 (0.86-1.21) | 0.96 (0.80-1.14) | 0.436 | 0.643 |
| **Fully-adjusted models** ^a^ |  |  |  |  |  |  |
| No. of cases | 185 | 204 | 196 | 176 |  |  |
| Person-years | 70,986 | 72,574 | 74,118 | 72,911 |  |  |
| HR (95%CI) | 1.00 (ref) | 1.05 (0.86-1.29) | 0.98 (0.79-1.20) | 0.86 (0.69-1.07) | 0.096 | 0.280 |
| **Premenopausal Breast Cancer** |  |  |  |  |  |  |
| NEAC | <6.6 | 6.6-8.8 | 8.8-11.6 | ≥11.6 |  |  |
| **Age-adjusted models** |  |  |  |  |  |  |
| No. of cases | 31 | 29 | 29 | 34 |  |  |
| Person-years | 26,780 | 26,675 | 26,540 | 25,749 |  |  |
| HR (95%CI) | 1.00 (ref) | 0.97 (0.59-1.61) | 0.98 (0.59-1.63) | 1.15 (0.71-1.87) | 0.530 | 0.894 |
| **Fully-adjusted models** ^b^ |  |  |  |  |  |  |
| No. of cases | 22 | 23 | 23 | 31 |  |  |
| Person-years | 22,081 | 22,408 | 22,388 | 21,733 |  |  |
| HR (95%CI) | 1.00 (ref) | 1.08 (0.60-1.95) | 1.08 (0.60-1.96) | 1.54 (0.87-2.72) | 0.153 | 0.422 |
| **Postmenopausal Breast Cancer** |  |  |  |  |  |  |
| NEAC | <6.7 | 6.7-8.9 | 8.9-11.8 | ≥11.8 |  |  |
| **Age-adjusted models** |  |  |  |  |  |  |
| No. of cases | 215 | 239 | 232 | 203 |  |  |
| Person-years | 65,675 | 67,097 | 67,360 | 67,745 |  |  |
| HR (95%CI) | 1.00 (ref) | 1.08 (0.89-1.29) | 1.04 (0.86-1.25) | 0.90 (0.75-1.09) | 0.187 | 0.297 |
| **Fully-adjusted models** ^c^ |  |  |  |  |  |  |
| No. of cases | 160 | 175 | 178 | 139 |  |  |
| Person-years | 46,071 | 48,547 | 49,944 | 49,871 |  |  |
| HR (95%CI) | 1.00 (ref) | 1.01 (0.81-1.25) | 0.99 (0.79-1.23) | 0.74 (0.58-0.95) | 0.010 | 0.035 |

Abbreviations: CI=confidence interval, HR=hazard ratio.

^a^ Hazard ratios from fully-adjusted models for breast cancer overall are adjusted for: age, body mass index, menopausal status, energy intake, educational level, cigarette smoking status, alcohol drinking, coffee drinking, physical activity, vitamins and minerals use, contraceptive pill use, hormone replacement therapy, age at the first menstruation, number of children and childlessness. Participants with missing values in covariates are excluded from the models.

^b^ Hazard ratios from fully-adjusted models for premenopausal breast cancer are adjusted for: age, body mass index, energy intake, educational level, cigarette smoking status, alcohol drinking, coffee drinking, physical activity, vitamins and minerals use, contraceptive pill use, age at the first menstruation, number of children and childlessness. Participants with missing values in covariates are excluded from the models.

^c^ Hazard ratios from fully-adjusted models for postmenopausal breast cancer are adjusted for: age, body mass index, energy intake, educational level, cigarette smoking status, alcohol drinking, coffee drinking, physical activity, vitamins and minerals use, contraceptive pill use, hormone replacement therapy, age at the first menstruation, number of children and childlessness. Participants with missing values in covariates are excluded from the models.

**Supplementary Table 7. Hazard ratios of postmenopausal breast cancer for dietary NEAC intake by subgroups in the National March Cohort after exclusion of the first two years of follow-up.**

|  | Dietary NEAC | | | |  |  |
| --- | --- | --- | --- | --- | --- | --- |
| Stratification variable | <6.7 | 6.7-8.9 | 8.9-11.8 | ≥11.8 | *P* _trend_ | *P* _Wald_ |
| **Hormone replacement therapy** |  |  |  |  |  |  |
| *No* |  |  |  |  |  |  |
| No. of cases | 98 | 90 | 105 | 78 |  |  |
| Person-years | 31,564 | 31,848 | 32,490 | 30,797 |  |  |
| HR (95%CI) ^a^ | 1.00 (ref) | 0.88 (0.66-1.17) | 1.02 (0.77-1.35) | 0.79 (0.57-1.08) | 0.226 | 0.307 |
| *Yes* |  |  |  |  |  |  |
| No. of cases | 62 | 85 | 73 | 61 |  |  |
| Person-years | 14,508 | 16,699 | 17,454 | 19,074 |  |  |
| HR (95%CI) ^a^ | 1.00 (ref) | 1.19 (0.85-1.65) | 0.98 (0.69-1.38) | 0.70 (0.48-1.01) | 0.014 | 0.027 |
| **Vitamins and minerals use** |  |  |  |  |  |  |
| *No* |  |  |  |  |  |  |
| No. of cases | 98 | 114 | 117 | 90 |  |  |
| Person-years | 30,093 | 31,729 | 31,910 | 32,612 |  |  |
| HR (95%CI) ^a^ | 1.00 (ref) | 1.07 (0.82-1.41) | 1.07 (0.81-1.41) | 0.75 (0.55-1.02) | 0.041 | 0.058 |
| *Yes* |  |  |  |  |  |  |
| No. of cases | 62 | 61 | 61 | 49 |  |  |
| Person-years | 15,979 | 16,818 | 18,034 | 17,259 |  |  |
| HR (95%CI) ^a^ | 1.00 (ref) | 0.92 (0.64-1.32) | 0.88 (0.61-1.27) | 0.72 (0.49-1.08) | 0.109 | 0.455 |
| **Cigarette smoking** |  |  |  |  |  |  |
| *Former or never* |  |  |  |  |  |  |
| No. of cases | 135 | 157 | 165 | 130 |  |  |
| Person-years | 40,688 | 44,578 | 46,451 | 47,254 |  |  |
| HR (95%CI) ^a^ | 1.00 (ref) | 1.03 (0.82-1.30) | 1.02 (0.81-1.29) | 0.76 (0.59-0.98) | 0.023 | 0.041 |
| *Current* |  |  |  |  |  |  |
| No. of cases | 25 | 18 | 13 | 9 |  |  |
| Person-years | 5,383 | 3,969 | 3,493 | 2,618 |  |  |
| HR (95%CI) ^a^ | 1.00 (ref) | 0.93 (0.50-1.73) | 0.77 (0.38-1.54) | 0.61 (0.27-1.37) | 0.192 | 0.635 |
| **Age** |  |  |  |  |  |  |
| *55-70 years* |  |  |  |  |  |  |
| No. of cases | 66 | 96 | 88 | 65 |  |  |
| Person-years | 18,505 | 22,089 | 21,538 | 22,033 |  |  |
| HR (95%CI) ^a^ | 1.00 (ref) | 1.21 (0.88-1.67) | 1.14 (0.82-1.57) | 0.79 (0.56-1.13) | 0.040 | 0.055 |
| *>=70 years* |  |  |  |  |  |  |
| No. of cases | 13 | 12 | 13 | 13 |  |  |
| Person-years | 3,086 | 3,889 | 4,504 | 4,443 |  |  |
| HR (95%CI) ^a^ | 1.00 (ref) | 0.86 (0.38-1.92) | 0.68 (0.31-1.51) | 0.74 (0.33-1.67) | 0.210 | 0.800 |

Abbreviations: CI=confidence interval, HR=hazard ratio.

^a^ Fully-adjusted hazard ratios for postmenopausal breast cancer are adjusted by: body mass index energy intake, educational level, cigarette smoking status, alcohol drinking, coffee drinking, physical activity, vitamins and minerals use, contraceptive pill use, hormone replacement therapy, age at the first menstruation, number of children and childlessness. Participants with missing values in covariates are excluded from the models.

**Supplementary Table 8. Hazard ratios of breast cancer for dietary NEAC intake from fruits and vegetables in the National March Cohort after exclusion of the first two years of follow-up.**

| Outcome | Dietary NEAC from Fruits and Vegetables | | | | *P* _trend_ | *P* _Wald_ |
| --- | --- | --- | --- | --- | --- | --- |
| **Breast Cancer** |  |  |  |  |  |  |
| NEAC | <1.3 | 1.3- 2.0 | 2.0-2.9 | ≥2.9 |  |  |
| **Age-adjusted models** |  |  |  |  |  |  |
| No. of cases | 252 | 257 | 268 | 254 |  |  |
| Person-years | 96,910 | 96,740 | 95,801 | 94,834 |  |  |
| HR (95%CI) | 1.00 (ref) | 0.91 (0.76-1.08) | 0.91 (0.77-1.07) | 0.83 (0.70-0.99) | 0.057 | 0.252 |
| **Fully-adjusted models** ^a^ |  |  |  |  |  |  |
| No. of cases | 182 | 199 | 211 | 168 |  |  |
| Person-years | 73,276 | 73,342 | 73,201 | 70,496 |  |  |
| HR (95%CI) | 1.00 (ref) | 0.95 (0.77-1.16) | 0.95 (0.77-1.16) | 0.75 (0.60-0.93) | 0.008 | 0.040 |
| **Premenopausal Breast Cancer** |  |  |  |  |  |  |
| NEAC | <1.2 | 1.2-1.8 | 1.8-2.6 | ≥2.6 |  |  |
| **Age-adjusted models** |  |  |  |  |  |  |
| No. of cases | 42 | 30 | 27 | 24 |  |  |
| Person-years | 30,982 | 26,629 | 24,679 | 23,274 |  |  |
| HR (95%CI) | 1.00 (ref) | 0.73 (0.46-1.16) | 0.68 (0.42-1.11) | 0.64 (0.39-1.06) | 0.090 | 0.249 |
| **Fully-adjusted models** ^b^ |  |  |  |  |  |  |
| No. of cases | 33 | 26 | 21 | 19 |  |  |
| Person-years | 26,029 | 22,110 | 21,007 | 19,382 |  |  |
| HR (95%CI) | 1.00 (ref) | 0.80 (0.48-1.35) | 0.66 (0.38-1.15) | 0.64 (0.36-1.14) | 0.111 | 0.360 |
| **Postmenopausal Breast Cancer** |  |  |  |  |  |  |
| NEAC | <1.4 | 1.4-2.1 | 2.1-2.9 | ≥2.9 |  |  |
| **Age-adjusted models** |  |  |  |  |  |  |
| No. of cases | 219 | 227 | 227 | 215 |  |  |
| Person-years | 62,737 | 66,479 | 67,838 | 70,286 |  |  |
| HR (95%CI) | 1.00 (ref) | 0.97 (0.80-1.17) | 0.94 (0.78-1.14) | 0.85 (0.70-1.03) | 0.079 | 0.365 |
| **Fully-adjusted models** ^c^ |  |  |  |  |  |  |
| No. of cases | 154 | 180 | 177 | 140 |  |  |
| Person-years | 44,477 | 48,722 | 50,217 | 50,833 |  |  |
| HR (95%CI) | 1.00 (ref) | 1.05 (0.84-1.30) | 0.98 (0.79-1.22) | 0.76 (0.61-0.96) | 0.009 | 0.031 |

Abbreviations: CI=confidence interval, HR=hazard ratio.

^a^ Hazard ratios from fully-adjusted models for breast cancer overall are adjusted for: age, body mass index, menopausal status, energy intake, educational level, cigarette smoking status, alcohol drinking, coffee drinking, physical activity, vitamins and minerals use, contraceptive pill use, hormone replacement therapy, age at the first menstruation, number of children and childlessness. Participants with missing values in covariates are excluded from the models.

^b^ Hazard ratios from fully-adjusted models for premenopausal breast cancer are adjusted for: age, body mass index, energy intake, educational level, cigarette smoking status, alcohol drinking, coffee drinking, physical activity, vitamins and minerals use, contraceptive pill use, age at the first menstruation, number of children and childlessness. Participants with missing values in covariates are excluded from the models.

^c^ Hazard ratios from fully-adjusted models for postmenopausal breast cancer are adjusted for: age, body mass index, energy intake, educational level, cigarette smoking status, alcohol drinking, coffee drinking, physical activity, vitamins and minerals use, contraceptive pill use, hormone replacement therapy, age at the first menstruation, number of children and childlessness. Participants with missing values in covariates are excluded from the models.

**Supplementary Table 9. Hazard ratios of breast cancer for dietary NEAC intake from grains in the National March Cohort after exclusion of the first two years of follow-up.**

| Outcome | Dietary NEAC from Grains | | | | *P* _trend_ | *P* _Wald_ |
| --- | --- | --- | --- | --- | --- | --- |
| **Breast Cancer** |  |  |  |  |  |  |
| NEAC | <1.2 | 1.2-1.5 | 1.5-2.0 | ≥2.0 |  |  |
| **Age-adjusted models** |  |  |  |  |  |  |
| No. of cases | 226 | 275 | 260 | 267 |  |  |
| Person-years | 97,556 | 96,695 | 96,521 | 93,322 |  |  |
| HR (95%CI) | 1.00 (ref) | 1.17 (0.98-1.40) | 1.06 (0.89-1.27) | 1.03 (0.86-1.23) | 0.709 | 0.288 |
| **Fully-adjusted models** ^a^ |  |  |  |  |  |  |
| No. of cases | 170 | 193 | 199 | 196 |  |  |
| Person-years | 72,975 | 75,067 | 74,446 | 67,590 |  |  |
| HR (95%CI) | 1.00 (ref) | 1.05 (0.85-1.29) | 1.04 (0.85-1.28) | 1.06 (0.86-1.31) | 0.667 | 0.953 |
| **Premenopausal Breast Cancer** |  |  |  |  |  |  |
| NEAC | <1.1 | 1.1-1.4 | 1.4-1.8 | ≥1.8 |  |  |
| **Age-adjusted models** |  |  |  |  |  |  |
| No. of cases | 32 | 32 | 32 | 27 |  |  |
| Person-years | 27,659 | 26,937 | 25,845 | 25,193 |  |  |
| HR (95%CI) | 1.00 (ref) | 1.00 (0.61-1.62) | 1.00 (0.61-1.64) | 0.87 (0.52-1.45) | 0.585 | 0.939 |
| **Fully-adjusted models** ^b^ |  |  |  |  |  |  |
| No. of cases | 27 | 26 | 24 | 22 |  |  |
| Person-years | 22,615 | 22,761 | 22,029 | 21,113 |  |  |
| HR (95%CI) | 1.00 (ref) | 0.90 (0.52-1.54) | 0.83 (0.48-1.45) | 0.79 (0.44-1.39) | 0.403 | 0.853 |
| **Postmenopausal Breast Cancer** |  |  |  |  |  |  |
| NEAC | <1.2 | 1.2-1.5 | 1.5-2.1 | ≥2.1 |  |  |
| **Age-adjusted models** |  |  |  |  |  |  |
| No. of cases | 186 | 246 | 221 | 232 |  |  |
| Person-years | 64,830 | 65,504 | 67,167 | 69,593 |  |  |
| HR (95%CI) | 1.00 (ref) | 1.30 (1.07-1.57) | 1.12 (0.92-1.36) | 1.10 (0.91-1.34) | 0.968 | 0.058 |
| **Fully-adjusted models** ^c^ |  |  |  |  |  |  |
| No. of cases | 137 | 171 | 167 | 174 |  |  |
| Person-years | 46,316 | 49,144 | 49,835 | 48,735 |  |  |
| HR (95%CI) | 1.00 (ref) | 1.17 (0.94-1.47) | 1.11 (0.88-1.39) | 1.20 (0.96-1.51) | 0.202 | 0.397 |

Abbreviations: CI=confidence interval, HR=hazard ratio.

^a^ Hazard ratios from fully-adjusted models for breast cancer overall are adjusted for: age, body mass index, menopausal status, energy intake, educational level, cigarette smoking status, alcohol drinking, coffee drinking, physical activity, vitamins and minerals use, contraceptive pill use, hormone replacement therapy, age at the first menstruation, number of children and childlessness. Participants with missing values in covariates are excluded from the models.

^b^ Hazard ratios from fully-adjusted models for premenopausal breast cancer are adjusted for: age, body mass index, energy intake, educational level, cigarette smoking status, alcohol drinking, coffee drinking, physical activity, vitamins and minerals use, contraceptive pill use, age at the first menstruation, number of children and childlessness. Participants with missing values in covariates are excluded from the models.

^c^ Hazard ratios from fully-adjusted models for postmenopausal breast cancer are adjusted for: age, body mass index, energy intake, educational level, cigarette smoking status, alcohol drinking, coffee drinking, physical activity, vitamins and minerals use, contraceptive pill use, hormone replacement therapy, age at the first menstruation, number of children and childlessness. Participants with missing values in covariates are excluded from the models.

**Supplementary Table 10. Hazard ratios of breast cancer for dietary NEAC intake in the National March Cohort after restriction to ten years of follow-up.**

| Outcome | Dietary NEAC | | | | *P* _trend_ | *P* _Wald_ |
| --- | --- | --- | --- | --- | --- | --- |
| **Breast Cancer** |  |  |  |  |  |  |
| NEAC | <6.7 | 6.7- 8.9 | 8.9-11.7 | ≥11.7 |  |  |
| **Age-adjusted models** |  |  |  |  |  |  |
| No. of cases | 141 | 147 | 136 | 138 |  |  |
| Person-years | 59,849 | 59,817 | 59,765 | 59,589 |  |  |
| HR (95%CI) | 1.00 (ref) | 1.01 (0.80-1.28) | 0.94 (0.74-1.19) | 0.93 (0.74-1.18) | 0.457 | 0.863 |
| **Fully-adjusted models** ^a^ |  |  |  |  |  |  |
| No. of cases | 100 | 110 | 94 | 97 |  |  |
| Person-years | 43,535 | 44,774 | 45,565 | 44,987 |  |  |
| HR (95%CI) | 1.00 (ref) | 1.02 (0.78-1.34) | 0.84 (0.63-1.13) | 0.84 (0.63-1.14) | 0.159 | 0.390 |
| **Premenopausal Breast Cancer** |  |  |  |  |  |  |
| NEAC | <6.6 | 6.6-8.8 | 8.8-11.6 | ≥11.6 |  |  |
| **Age-adjusted models** |  |  |  |  |  |  |
| No. of cases | 22 | 18 | 14 | 27 |  |  |
| Person-years | 21,531 | 21,235 | 21,093 | 20,679 |  |  |
| HR (95%CI) | 1.00 (ref) | 0.86 (0.46-1.60) | 0.67 (0.34-1.31) | 1.29 (0.73-2.26) | 0.325 | 0.227 |
| **Fully-adjusted models** ^b^ |  |  |  |  |  |  |
| No. of cases | 15 | 14 | 12 | 26 |  |  |
| Person-years | 17,576 | 17,676 | 17,696 | 17,376 |  |  |
| HR (95%CI) | 1.00 (ref) | 1.94 (0.45-1.96) | 0.82 (0.38-1.77) | 1.72 (0.87-3.39) | 0.072 | 0.124 |
| **Postmenopausal Breast Cancer** |  |  |  |  |  |  |
| NEAC | <6.7 | 6.7-8.9 | 8.9-11.8 | ≥11.8 |  |  |
| **Age-adjusted models** |  |  |  |  |  |  |
| No. of cases | 146 | 156 | 149 | 125 |  |  |
| Person-years | 46,098 | 46,528 | 46,511 | 46,686 |  |  |
| HR (95%CI) | 1.00 (ref) | 1.04 (0.83-1.31) | 1.00 (0.80-1.26) | 0.83 (0.66-1.06) | 0.091 | 0.263 |
| **Fully-adjusted models** ^c^ |  |  |  |  |  |  |
| No. of cases | 105 | 118 | 107 | 85 |  |  |
| Person-years | 32,367 | 33,677 | 34,476 | 34,324 |  |  |
| HR (95%CI) | 1.00 (ref) | 1.04 (0.79-1.35) | 0.91 (0.69-1.21) | 0.71 (0.52-0.96) | 0.012 | 0.061 |

Abbreviations: CI=confidence interval, HR=hazard ratio.

^a^ Hazard ratios from fully-adjusted models for breast cancer overall are adjusted for: age, body mass index, menopausal status, energy intake, educational level, cigarette smoking status, alcohol drinking, coffee drinking, physical activity, vitamins and minerals use, contraceptive pill use, hormone replacement therapy, age at the first menstruation, number of children and childlessness. Participants with missing values in covariates are excluded from the models.

^b^ Hazard ratios from fully-adjusted models for premenopausal breast cancer are adjusted for: age, body mass index, energy intake, educational level, cigarette smoking status, alcohol drinking, coffee drinking, physical activity, vitamins and minerals use, contraceptive pill use, age at the first menstruation, number of children and childlessness. Participants with missing values in covariates are excluded from the models.

^c^ Hazard ratios from fully-adjusted models for postmenopausal breast cancer are adjusted for: age, body mass index, energy intake, educational level, cigarette smoking status, alcohol drinking, coffee drinking, physical activity, vitamins and minerals use, contraceptive pill use, hormone replacement therapy, age at the first menstruation, number of children and childlessness. Participants with missing values in covariates are excluded from the models.

**Supplementary Table 11. Hazard ratios of breast cancer for dietary NEAC intake in the National March Cohort after imputation of menopausal age.**

| Outcome | Dietary NEAC ^a^ | | | | *P* _trend_ | *P* _Wald_ |
| --- | --- | --- | --- | --- | --- | --- |
| **Premenopausal Breast Cancer** |  |  |  |  |  |  |
| NEAC | <6.6 | 6.6-8.8 | 8.8-11.6 | ≥11.6 |  |  |
| **Age-adjusted models** |  |  |  |  |  |  |
| No. of cases | 10 | 15 | 10 | 15 |  |  |
| Person-years | 22,104 | 22,282 | 22,522 | 21,375 |  |  |
| HR (95%CI) | 1.00 (ref) | 1.54 (0.69-3.43) | 0.99 (0.41-2.38) | 1.54 (0.69-3.42) | 0.387 | 0.519 |
| **Fully-adjusted models** ^b^ |  |  |  |  |  |  |
| No. of cases | 7 | 12 | 9 | 14 |  |  |
| Person-years | 18,196 | 18,737 | 18,973 | 18,050 |  |  |
| HR (95%CI) | 1.00 (ref) | 1.82 (0.71-4.65) | 1.20 (0.44-3.26) | 1.81 (0.70-4.66) | 0.316 | 0.481 |
| **Postmenopausal Breast Cancer** |  |  |  |  |  |  |
| NEAC | <6.7 | 6.7-8.9 | 8.9-11.8 | ≥11.8 |  |  |
| **Age-adjusted models** |  |  |  |  |  |  |
| No. of cases | 234 | 261 | 256 | 223 |  |  |
| Person-years | 85,051 | 86,163 | 86,075 | 86,419 |  |  |
| HR (95%CI) | 1.00 (ref) | 1.07 (0.90-1.28) | 1.05 (0.88-1.25) | 0.90 (0.75-1.09) | 0.232 | 0.255 |
| **Fully-adjusted models** ^c^ |  |  |  |  |  |  |
| No. of cases | 173 | 194 | 194 | 152 |  |  |
| Person-years | 60,302 | 63,043 | 64,262 | 64,288 |  |  |
| HR (95%CI) | 1.00 (ref) | 1.03 (0.84-1.27) | 1.00 (0.81-1.23) | 0.75 (0.59-0.94) | 0.011 | 0.020 |

Abbreviations: CI=confidence interval, HR=hazard ratio.

^a^ NEAC was measured with the NEAC assay expressed in mmol Fe2+ equivalents/day.

^b^ Hazard ratios from fully-adjusted models for premenopausal breast cancer are adjusted for: age, body mass index, energy intake, educational level, cigarette smoking status, alcohol drinking, coffee drinking, physical activity, vitamins and minerals use, contraceptive pill use, age at the first menstruation, number of children and childlessness. Participants with missing values in covariates are excluded from the models.

^c^ Hazard ratios from fully-adjusted models for postmenopausal breast cancer are adjusted for: age, body mass index, energy intake, educational level, cigarette smoking status, alcohol drinking, coffee drinking, physical activity, vitamins and minerals use, contraceptive pill use, hormone replacement therapy, age at the first menstruation, number of children and childlessness. Participants with missing values in covariates are excluded from the models.
